# Oxygen therapy and noninvasive respiratory supports in acute hypoxemic respiratory failure: a narrative review

**DOI:** 10.1186/s13613-024-01389-w

**Published:** 2024-10-18

**Authors:** Arnaud W. Thille, Frédéric Balen, Guillaume Carteaux, Tahar Chouihed, Jean-Pierre Frat, Christophe Girault, Erwan L’Her, Nicolas Marjanovic, Mai-Anh Nay, Patrick Ray, Matthieu Reffienna, Leo Retenauer, Antoine Roch, Guillaume Thiery, Jennifer Truchot

**Affiliations:** 1 Service de Médecine Intensive Réanimation, CHU de Poitiers, Poitiers, France; 2 INSERM CIC-1402, IS- ALIVE, Université de Poitiers, Poitiers, France; 3 CHU de Toulouse, Service des Urgences, Toulouse, France; 4 INSERM, CERPOP – EQUITY, Toulouse, France; 5 Assistance Publique-Hôpitaux de Paris, CHU Henri Mondor-Albert Chenevier, Service de Médecine Intensive Réanimation, Créteil, France; 6 Faculté de Santé, Groupe de Recherche Clinique CARMAS, Université Paris Est-Créteil, Créteil, France; 7 INSERM U955, Institut Mondor de Recherche Biomédicale, Créteil, France; 8 CHRU de Nancy, Service des Urgences, Nancy, France; 9 Université de Lorraine, UMRS 1116, Nancy, France; 10 CHU-Hôpitaux de Rouen, Service de Médecine Intensive Réanimation, Normandie Univ, GRHVN UR, Rouen, 3830 France; 11 CHU de Brest, Service de Médecine Intensive Réanimation, Brest, France; 12 CHU de Poitiers, Service d’Accueil des Urgences, Poitiers, France; 13 CHU d’Orléans, Service de Médecine Intensive Réanimation, Orléans, France; 14 CHU de Dijon, Service des Urgences, Dijon, France; 15 Hôpital Foch, Service de Réanimation polyvalente, Suresnes, France; 16 Assistance Publique-Hôpitaux de Paris, Hôpital de la Pitié-Salpêtrière, Service des Urgences, Paris, France; 17 CHU de Marseille, Hôpital Nord, Service de Médecine Intensive Réanimation, Marseille, France; 18 CHU de Saint-Etienne, Service de Médecine Intensive Réanimation, Saint-Etienne, France; 19 Research on Healthcare Performance RESHAPE, INSERM U1290, Université Claude Bernard Lyon 1, Lyon, France; 20 Assistance Publique – Hôpitaux de Paris, Hôpital Cochin, Service des Urgences, Université Paris-Cité, Paris, France

## Abstract

**Background:**

This narrative review was written by an expert panel to the members of the jury to help in the development of clinical practice guidelines on oxygen therapy.

**Results:**

According to the expert panel, acute hypoxemic respiratory failure was defined as PaO_2_ < 60 mm Hg or SpO_2_ < 90% on room air, or PaO_2_/FiO_2_ ≤ 300 mm Hg. Supplemental oxygen should be administered according to the monitoring of SpO_2_, with the aim at maintaining SpO_2_ above 92% and below 98%. Noninvasive respiratory supports are generally reserved for the most hypoxemic patients with the aim of relieving dyspnea. High-flow nasal cannula oxygen (HFNC) seems superior to conventional oxygen therapy (COT) as a means of avoiding intubation and may therefore be should probably be used as a first-line noninvasive respiratory support in patients requiring more than 6 L/min of oxygen or PaO_2_/FiO_2_ ≤ 200 mm Hg and a respiratory rate above 25 breaths/minute or clinical signs of respiratory distress, but with no benefits on mortality. Continuous positive airway pressure (CPAP) cannot currently be recommended as a first-line noninvasive respiratory support, since its beneficial effects on intubation remain uncertain. Despite older studies favoring noninvasive ventilation (NIV) over COT, recent clinical trials fail to show beneficial effects with NIV compared to HFNC. Therefore, there is no evidence to support the use of NIV or CPAP as first-line treatment if HFNC is available. Clinical trials do not support the hypothesis that noninvasive respiratory supports may lead to late intubation. The potential benefits of awake prone positioning on the risk of intubation in patients with COVID-19 cannot be extrapolated to patients with another etiology.

**Conclusions:**

Whereas oxygen supplementation should be initiated for patients with acute hypoxemic respiratory failure defined as PaO_2_ below 60 mm Hg or SpO_2_ < 90% on room air, HFNC should be the first-line noninvasive respiratory support in patients with PaO_2_/FiO_2_ ≤ 200 mm Hg with increased respiratory rate. Further studies are needed to assess the potential benefits of CPAP, NIV through a helmet and awake prone position in patients with acute hypoxemic respiratory failure not related to COVID-19.

## Definition of acute hypoxemic respiratory failure

Respiratory failure is defined as the failure of the respiratory system to perform its gas exchange functions, including oxygenation and carbon dioxide elimination. Acute hypoxemic respiratory failure, also referred to as type 1, specifically denotes oxygenation failure and refers to *de novo* respiratory failure, i.e. excluding hypoxemia caused by cardiogenic pulmonary edema and exacerbation of chronic lung diseases. The main etiology of such *de novo* respiratory failure is pneumonia [1]. Thus, acute hypoxemic respiratory failure does not encompass acute hypercapnic respiratory failure, also referred to as type 2, characterized by a ventilation failure defined by arterial carbon dioxide pressure (PaCO_2_) exceeding 45 mm Hg and pH below 7.35, indicative of respiratory acidosis. This review will not address the latter form of respiratory failure.

According to the panel of experts participating in these guidelines on oxygen therapy [2], acute hypoxemic respiratory failure can be defined as partial pressure of arterial oxygen (PaO_2_) less than 60 mm Hg or peripheral oxygen saturation measured by pulse oximetry (SpO_2_) less than 90% on room air, or a ratio of the partial pressure of arterial oxygen to the fraction of inspired oxygen (PaO_2_/FiO_2_) of 300 mm Hg or less ratio less in patients receiving oxygen therapy. Whereas FiO_2_ is not measured under conventional oxygen therapy (COT) delivered through nasal cannula or facemask, it can be best estimated using the following formula: FiO_2_ in % = Flow of oxygen (L/min) x 3 + 21% [3]. Regardless of the oxygenation strategy, hypoxemia can be considered as severe in patients with PaO_2_/FiO_2_ ratio equal to or below 100 mm Hg, moderate in patients with PaO_2_/FiO_2_ ratio between 101 and 200 mm Hg, and mild in patients with PaO_2_/FiO_2_ ratio between 201 and 300 mm Hg, as in patients with acute respiratory distress syndrome (ARDS) [4]. Clinical signs of acute hypoxemic respiratory failure include increased respiratory rate above 25 breaths per minute, activation of accessory respiratory muscles, paradoxical abdominal respiration, cyanosis, dyspnea, and breathlessness. Several oxygenation strategies can be proposed as alternatives to COT for oxygen supplementation, including high-flow nasal cannula oxygen therapy (HFNC), continuous positive airway pressure (CPAP), and noninvasive ventilation (NIV). Among patients with acute hypoxemic respiratory failure, a vast majority have pulmonary bilateral infiltrates on chest radiograph and could be considered as having ARDS criteria [1, 5]. However, as the Berlin definition of ARDS stated that PaO_2_/FiO_2_ ratio had to be measured with positive airway pressure of at least 5 cmH_2_O [4], patients treated with COT or HFNC still cannot be considered as ARDS [6]. Indeed, positive airway pressure levels generated with HFNC are most often around 2–3 cm H_2_O and remain lower than the levels required to reach ARDS criteria [7, 8]. To modify this definition, recent guidelines suggested considering patients treated with HFNC at a minimal 30 L/min flow as having ARDS if they have pulmonary bilateral infiltrates and PaO_2_/FiO_2_ ratio of 300 mm Hg or less [9].

## Indications and targets for oxygen therapy

### Deleterious effects of hypoxemia

While the deleterious effects of hypoxemia are widely known in clinical practice, this concept has been poorly evaluated. In a large retrospective cohort study including 152,680 patients admitted to 150 ICUs, hypoxemia occurring within the first 24 h following admission was associated with an increased risk of death [10]. The same findings were also reported in a systematic review including 7,410 critically ill children [11]. A more recent multicenter clinical trial comparing conservative oxygen therapy (PaO_2_ between 55 and 70 mm Hg and SpO_2_ between 88 and 92%) versus liberal oxygen therapy (PaO_2_ between 90 and 105 mm Hg and SpO_2_ of at least 96%) in mechanically ventilated ARDS patients was prematurely stopped after including 205 patients because of safety concerns [12]. Indeed, mortality was significantly higher in the arm receiving the lower oxygen therapy strategy. These findings lend credence to an oxygen administration strategy that aims at avoiding hypoxemia by maintaining PaO_2_ above 70 mm Hg and SpO_2_ above 92% in patients with acute hypoxemic respiratory failure, even though most of the data come from mechanically ventilated patients.

### Deleterious effects of hyperoxia

Animal studies have consistently shown that exposure to high FiO_2_ can cause respiratory failure and early death [13]. From the beginnings of artificial ventilation, there has been concern that high FiO_2_ values might induce pulmonary lesions [14]. Whereas ventilator-induced lung injury (VILI) is well- established [15], the specific pulmonary lesions attributable to oxygen toxicity remain hypothetical. Literature from the past 15 years has yielded extensive yet conflicting findings. The challenge in interpreting the different clinical trials stems from their varied methodologies while some focused exclusively on mechanically ventilated patients, while others included any patients breathing spontaneously under oxygen. Additionally, oxygenation targets have varied, with some studies using SpO_2_ and others PaO_2_, often with significant overlap between the two targets (Table 1). A first seminal single-center clinical trial including 434 unselected patients admitted to an ICU showed that a higher oxygen strategy (SpO_2_ between 97 and 100%) significantly increased the risk of death as compared to a lower oxygen strategy (SpO_2_ between 94 and 98%) [16]. To date, this is the only randomized controlled trial showing increased risk of death with a higher oxygen strategy and subsequent clinical trials have found no significant differences in mortality between lower and higher oxygenation strategies [17–25]. Nevertheless, a recent clinical trial showed the potentially deleterious effects of higher oxygen strategy, with a decreased number of days without life support in COVID-19 patients admitted to ICUs [17]. Furthermore, a separate clinical trial revealed that administration of maximal FiO_2_ (set at 100% for 24 h) in mechanically ventilated patients with septic shock led to a higher incidence of serious adverse events and a trend toward increased mortality, prompting the study to be stopped due to safety concerns [26]. Additionally, in a large retrospective cohort study including 19,593 patients admitted to five ICUs in the United Kingdom, exposure to hyperoxia (defined as PaO_2_ ≥ 100 mm Hg), no matter its duration, increased risk of death when compared to patients non-exposed to hyperoxia [27]. Nevertheless, even though hyperoxia may have deleterious effects in critically ill patients, a recent meta-analysis pooling RCTs comparing higher versus lower oxygenation strategies suggested that it was still not possible to draw definitive conclusions about the effects on mortality [28]. Only studies targeting particularly high oxygenation levels have shown harmful effects on mortality [16, 26]. These findings support an oxygen administration strategy that aims at avoiding hyperoxia by maintaining PaO_2_ below 90–100 mm Hg and SpO_2_ below 98%.

**Table 1:** Main RCTs comparing different targets of oxygenation

Study, year *N* patients – centers	Targets PaO_2_ (mm Hg) – SpO_2_ (%) higher vs. lower	Inclusion criteria: % of intubated patients	Mortality rates
Nielsen, 2024 [17] *N* = 726–13 centers	High PaO_2_ 90 mm Hg vs. Low PaO_2_ 60	COVID-19: 24%	*Mortality day-90*: higher 35% vs. lower 30%, *p* = 0.18
Van der Wal, 2023 [18]; *N* = 664–9 centers	High PaO_2_ 110–150 mm Hg, SpO_2_ 96–100% vs. Low PaO_2_ 55–80, SpO_2_ 91–94%	Patients receiving MV > 24 h : 100%	*Mortality day-28*: higher 35% vs. lower 39%, *p* = 0.34
Schmidt, 2022 [19] *N* = 789–2 centers	High PaO_2_ 98–105 mm Hg vs. Low PaO_2_ 68–75	After cardiac arrest: 100%	*Mortality day-90*: higher 29% vs. lower 31%, p = NS
Semler, 2022 [20] *N* = 2541–1 center	High SpO_2_ 98% (96–100) vs. Intermediate SpO_2_ 94% (92–96) vs. Low SpO_2_ 90% (88–92)	Patients receiving MV: 100%	*Mortality day-28*: higher 33% vs. intermediate 34% vs. lower 35%, p = NS
Gelissen, 2021 [22] *N* = 574–4 centers	Higher PaO_2_ 105–135 mm Hg vs. Lower: PaO_2_ 60–90	SIRS : 70%	*Mortality day-90*: higher 34% vs. lower 35%, *p* = 0.91
Mackle, 2020 [23] *N* = 1000–14 centers	High SpO_2_ > 90% vs. Low SpO_2_ > 90% and < 97%	Patients receiving MV: 100%	*Mortality day-90*: higher 33% vs. lower 35%, p = NS
Schjørring, 2020 [21] *N* = 2928–35 centers	High PaO_2_ 90 mm Hg vs. Low PaO_2_ 60	Patients with oxygen (> 10 L/min): 59%	*Mortality day-90*: higher 42% vs. lower 43%, *p* = 0.60
Barrot, 2020 [12] *N* = 201–13 centers	High PaO_2_ 90–105 mm Hg, SpO_2_ ≥ 96% vs. Low PaO_2_ 55–70, SpO_2_ 88–92%	Patients with ARDS: 100%	*Mortality day-90*: higher 30% vs. lower 44%, *p* < 0.05 *Stopped for safety reason
Yang, 2019 [24] *N* = 14–1 center	Higher PaO_2_ 105–135 mm Hg vs. Lower PaO_2_ 60–90	Expected ICU stay > 72 h: 85%	*Mortality day-28*: higher 33% vs. lower 26%, *p* = 0.30
Asfar, 2017 [26] *N* = 442–22 centers	High FiO_2_ 100% for 24 h vs. Low SpO_2_ 88–95%	Septic shock: 100%	*Mortality day-28*: higher 43% vs. lower 35%, *p* = 0.12 *Stopped for safety reason
Panwar, 2016 [25] *N* = 103–4 centers	Higher SpO_2_ ≥ 96% vs. Lower SpO_2_ 88–92%	Patients receiving MV > 24 h : 100%	*Mortality day-90*: higher 37% vs. lower 40%, *p* = 0.74
Girardis, 2016 [16] *N* = 434–1 center	High PaO_2_ up to 150 mm Hg, SpO_2_ 97–100 vs. Low PaO_2_ 70–100, SpO_2_ 94–98	Expected ICU stay > 72 h: 67%	*Mortality ICU*: higher 20% vs. lower 12%, *p* = 0.01

**Abbreviations**: RCT = Randomized Controlled Trials; PaO_2_ = Partial pressure of arterial oxygen; SpO_2_ = peripheral oxygen saturation measured by pulse oximetry; FiO_2_ = Fraction of inspired oxygen

### Indications for oxygen therapy

Supplemental oxygen is frequently administered in emergency departments (EDs) and more than half of patients admitted to ICUs receive oxygen therapy for hypoxemia [29]. Oxygen is a treatment for hypoxemia, not breathlessness, and supplemental oxygen does not improve dyspnea and breathlessness in non-hypoxemic patients [30, 31]. Supplemental oxygen should be administered in patients with acute hypoxemic respiratory failure according to the monitoring of SpO_2_ values. In case of severe hypoxemia, oxygen therapy should be immediately administered using a non-rebreathing reservoir mask at a flow rate of at least 15 L/min. For less severe cases, oxygen should be administered using nasal prongs when the flow rate is between 1 and 6 L/min, or using a standard facemask when the flow rate is between 6 and 10 L/min. To avoid deleterious effects of hypoxemia and hyperoxia, SpO_2_ values should be maintained above 92% and below 98%. However, technical problems and measurement biases, such as low peripheral blood flow, anemia, and skin pigmentation can significantly compromise the accuracy of SpO_2_ measurements [32–34]. Moreover, there may be considerable discrepancies in SpO_2_ measurements between different monitoring devices, with errors ranging from − 3% to + 1% relative to arterial oxygen saturation (SaO_2_) as the reference [35].

## Noninvasive respiratory supports for management of respiratory failure

Several oxygenation strategies can be proposed as an alternative to COT in patients with acute hypoxemic respiratory failure. These oxygenation strategies, most often referred as noninvasive respiratory supports because of positive airway pressure and relief of respiratory effort include HFNC, CPAP, and NIV. Whereas supplemental oxygen should be offered to all patients with acute hypoxemic respiratory failure regardless of the level of breathlessness, noninvasive respiratory supports are reserved for the most hypoxemic patients with clinical signs of respiratory distress and are aimed at relieving dyspnea. In most clinical trials assessing noninvasive respiratory supports in acute hypoxemic respiratory failure, the patients included had moderate-to-severe hypoxemia (PaO_2_/FiO_2_ ≤ 200 mm Hg), and either an increased respiratory rate above 25 breaths per minute or clinical signs of respiratory distress (Tables 2 and 3, and 4). These operational criteria could enable identification of the most severe forms of acute hypoxemic respiratory failure, and help in deciding when to initiate a noninvasive respiratory support.

**Table 2:** Main RCTs comparing high-flow nasal cannula oxygen therapy (HFNC) vs. conventional oxygen therapy (COT) in acute hypoxemic respiratory failure

Study, year *N* centers	*N* Patients	HFNC vs. COT	Outcomes
Crimi, 2023 [51] 27 centers – wards	*N* = 362 – Mild-to-moderate COVID-19	HFNC (*n* = 181) vs. COT (*n* = 182)	*Intubation day-28*: HFNC 7% vs. COT 10% (p = NS) – Timing not specified *Mortality day-28*: HFNC 8% vs. COT 7% (*p* = 0.84)
Bouadma, 2022 [50] 19 centers – ICU	*N* = 333 –COVID-19	HFNC (*n* = 115) vs. COT (*n* = 109) vs. CPAP (*n* = 109)	*Intubation day-28*: HFNC 33% vs. COT 29% vs. CPAP 31% (p = NS) – Timing not specified *Mortality day-60*: HFNC 26% vs. COT 29% vs. CPAP 28% (p = NS)
Nazir, 2022 [52] 1 center – ICU	*N* = 120 - Mild-to-moderate COVID-19	HFNC [60] vs. COT (*n* = 60)	*Intubation*: HFNC 3% vs. COT 13% (p = NS) – Timing not specified *Mortality*: HFNC 5% vs. COT 8% (p = NS)
Perkins, 2022 [49] 48 centers – ICUs and wards	*N* = 783 – COVID-19	HFNC (415) vs. COT (*n* = 368)	*Intubation day-30*: HFNC 41% vs. COT 42% (*p* = 0.86) – Timing: HFNC 1 day [0–3] vs. COT 1 day [0–3] (*p* = 0.82) *Mortality day-30*: HFNC 19% vs. COT 20% (*p* = 0.66)
Frat, 2022 [48] 34 centers – ICU	*N* = 711 – COVID-19	HFNC (*n* = 357) vs. COT (*n* = 354)	*Intubation day-28*: HFNC 45% vs. COT 53% (*p* = 0.04) – Timing: HFNC 36 h [12–84] vs. COT 26 h [12–54] (*p* = 0.10) *Mortality ICU*: HFNC 10% vs. COT 11% (*p* = 0.60)
Ospina-Tascón, 2021 [47] 3 centers – ICU	*N* = 199 – COVID-19	HFNC (*n* = 99) vs. COT (*n* = 100)	*Intubation day-28*: HFNC 34% vs. COT 51% (*p* = 0.03) – Timing: HFNC 22 h [13–60] vs. COT 29 h [14–58] (*p* = 0.69) *Mortality day-28*: HFNC 8% vs. COT 16% (*p* = 0.11)
Andino, 2020 [42] 1 center – ICU	*N* = 46 – Acute hypoxemic respiratory failure	HFNC (*n* = 24) vs. COT (*n* = 24)	*Intubation*: HFNC 33% vs. COT 63% (*p* = 0.04) – Timing not specified *Mortality*: HFNC 25% vs. COT 18% (*p* = 0.70)
Azoulay, 2018 [41] 32 centers – ICU	*N* = 776 – Immuno-compromised	HFNC (*n* = 388) vs. COT (*n* = 388)	*Intubation*: HFNC 39% vs. COT 44% (*p* = 0.17) – Timing not specified *Mortality day-28*: HFNC 36% vs. COT 36% (*p* = 0.94)
Frat, 2015 [1] 24 centers – ICU	*N* = 310 – Acute hypoxemic respiratory failure	HFNC (*n* = 106) vs. COT (*n* = 94) vs. NIV (*n* = 110)	*Intubation day-28*: HFNC 38% vs. COT 47% vs. NIV 50% (*p* = 0.18) – Timing: HFNC 27 h [8–46] vs. COT 15 h [5–39] vs. NIV 27 h [8–53] (*p* = 0.27) *Mortality ICU*: HFNC 11% vs. COT 19% vs. NIV 25% (*p* = 0.047)

Values are given in mean ± standard deviation or median [25^ème^ – 75^ème^ percentiles]

**Abbreviations**: RCT = Randomized Controlled Trials; COT = Conventional Oxygen Therapy; HFNC = High-Flow Nasal Cannula oxygen therapy; CPAP = Continuous Positive Airway Pressure; ICU = Intensive Care Unit; NS = Not Significant

**Table 3:** Main RCTs comparing CPAP vs. COT or HFNC in acute hypoxemic respiratory failure

Study, year *N* centers	*N* Patients	CPAP vs. COT or HFNC	Outcomes
Bouadma, 2022 [50] 19 centers – ICU	*N* = 333; COVID-19	CPAP facemask (*n* = 109) vs. COT (*n* = 109) vs. HFNC (*n* = 115)	*Intubation day-28*: CPAP 31% vs. COT 29% vs. HFNC 33% (p = NS) – Timing not specified *Mortality day-60*: CPAP 28% vs. COT 29% vs. HFNC 26% (p = NS)
Perkins, 2022 [49] 48 centers – ICUs and wards	*N* = 733; COVID-19	CPAP facemask (*n* = 377) vs. COT (*n* = 356)	*Intubation day-30*: CPAP 33% vs. COT 41% (*p* = 0.03) – Timing: CPAP 2 days [1–4] vs. COT 1 day [0–4] (*p* = 0.03) *Mortality day-30*: CPAP 17% vs. COT 19% (*p* = 0.65)
Brambilla, 2014 [59] 4 centers	*N* = 81; Pneumonia	CPAP helmet (*n* = 40) vs. COT (*n* = 41)	*Intubation*: CPAP 5% vs. COT 2% (p = NS) – Timing not specified *Mortality hospital*: CPAP 5% vs. 17% (*p* = 0.15)
Squadrone, 2010 [61] 1 center – wards	*N* = 40; Hematologic with respiratory failure	CPAP helmet (*n* = 20) vs. COT (*n* = 20)	*Intubation*: CPAP 10% vs. COT 70% (*p* < 0.01) – Timing not specified *Mortality hospital*: CPAP 15% vs. 25% (*p* < 0.01)
Cosentini, 2010 [60] 1 center – wards	*N* = 47; Pneumonia	CPAP helmet (*n* = 20) vs. COT (*n* = 27)	No patient was intubated or died
Delclaux, 2000 [58] 6 centers – ICU	*N* = 123; Acute hypoxemic respiratory failure	CPAP facemask (*n* = 62) vs. COT (*n* = 61)	*Intubation*: CPAP 34% vs. COT 39% (*p* = 0.53) – Timing not specified *Mortality ICU*: CPAP 21% vs. COT 25% (*p* = 0.63)

Values are given in mean ± standard deviation or median [25^ème^ – 75^ème^ percentiles]

**Abbreviations**: RCT = Randomized Controlled Trials; ICU = Intensive Care Unit; ED = Emergency Department; RF = Respiratory Failure; ARDS = Acute Respiratory Distress Syndrome; COPD = Chronic Obstructive Pulmonary Disease; CPE = Cardiogenic Pulmonary Edema; COT = Conventional Oxygen Therapy; HFNC = High-Flow Nasal Cannula oxygen therapy; NIV = Non-Invasive Ventilation; NS = Not Significant

**Table 4:** Main RCTs comparing NIV vs. COT or HFNC in acute hypoxemic respiratory failure

Study, year *N* centers	*N* Patients	NIV vs. COT or HFNC	Outcomes
Coudroy, 2022 [78] 29 ICUs	*N* = 299; Immuno- Compromised – COPD/CPE: No	NIV facemask + HFNC (*n* = 145) vs. HFNC (*n* = 154)	*Intubation day-28*: NIV 46% vs. HFNC 51% (*p* = 0.44) – Timing: NIV 29 h [9–72] vs. HFNC 20 h [5–58] (*p* = 0.24) *Mortality day-28*: NIV 35% vs. 36% (*p* = 0.83)
Grieco, 2021 [79] 4 ICUs	*N* = 109; COVID-19 – COPD/CPE: No	NIV helmet (*n* = 55) vs. HFNC (*n* = 54)	*Intubation*: NIV 30% vs. HFNC 51% (*p* = 0.03) – Timing: NIV 29 h [8–71] vs. HFNC 21 h [4–65] (*p* = 0.45) *Mortality day-28*: NIV 15% vs. 18% (*p* = 0.80)
Nair, 2021 [77] 1 ICU	*N* = 109; COVID-19 – COPD/CPE: No	NIV facemask (*n* = 54) vs. HFNC (*n* = 55)	*Intubation day-7*: NIV 46% (*n* = 25) vs. HFNC 27% (*n* = 15) (*p* = 0.045) – Timing not specified *Mortality hospital*: NIV 46% (*n* = 25) vs. 29% (*n* = 16) (*p* = 0.06)
He, 2019 [76] 21 ICUs	*N* = 200; Mild Acute Respiratory Distress Syndrome – COPD/CPE: No	NIV facemask (*n* = 102) vs. COT (*n* = 98)	*Intubation*: NIV 9% vs. COT 7% (*p* = 0.66) – Timing: NIV 4.7 ± 6.7 days vs. COT 2.6 ± 2.9 (*p* = 0.38) *Mortality ICU*: NIV 7% vs. 7% (*p* = 0.72)
Doshi, 2018 [75] 5 EDs	*N* = 204; Acute hypoxemic respiratory failure – COPD: 39%	NIV facemask (*n* = 100) vs. HFNC (*n* = 104)	*Intubation at 72 h*: NIV 13% vs. HFNC 7% (*p* = 0.13) – Timing: NIV 2.5 h [1.0-6.4] vs. HFNC 4.0 h [2.1–5.5] (p = NS)
Lemiale, 2015 [74] 28 ICUs	*N* = 374; Immuno- Compromised – COPD/CPE: No	NIV facemask ± HFNC (*n* = 191) vs. COT or HFNC (*n* = 183)	*Intubation day-28*: NIV 38% vs. COT or HFNC 45% (*p* = 0.20) – Timing not specified *Mortality day-28*: NIV 24% vs. 27% (*p* = 0.47)
Frat, 2015 [1] 24 ICUs	*N* = 310; Acute hypoxemic respiratory failure – COPD/CPE: No	NIV facemask (*n* = 110) vs. COT (*n* = 94) or HFNC (*n* = 106)	*Intubation day-28*: NIV 50% vs. COT 47% or HFNC 38% (*p* = 0.18) – Timing: NIV 27 h [8–53] vs. COT 15 h [5–39] or HFNC 27 h [8–46] (*p* = 0.27) *Mortality ICU*: NIV 25% vs. COT 19% or HFNC 11% (*p* = 0.047)
Zhan, 2012 [80] 10 ICUs	*N* = 40; Mild Acute Respiratory Distress Syndrome – COPD/CPE: No	NIV facemask (*n* = 21) vs. COT (*n* = 19)	*Intubation*: NIV 5% vs. COT 21% (*p* = 0.17) – Timing not specified *Mortality ICU*: NIV 5% vs. 26% (*p* = 0.08)
Ferrer, 2003 [68] 3 ICUs	*N* = 105; Acute hypoxemic respiratory failure – CPE: 29%	NIV facemask (*n* = 51) vs. COT (*n* = 54)	*Intubation*: NIV 25% vs. COT 52% (*p* = 0.03) – Timing not specified *Mortality ICU*: NIV 18% vs. 39% (*p* = 0.03)
Hilbert, 2001 [69] 1 ICU	*N* = 52 Immuno-compromised – COPD/CPE: No	NIV facemask (*n* = 26) vs. COT (*n* = 26)	*Intubation*: NIV 46% vs. COT 77% (*p* = 0.03) – Timing: NIV 63 ± 16 h vs. COT 51 ± 23 h (p = NS) *Mortality ICU*: NIV 38% vs. 69% (*p* = 0.03)
Antonelli, 2000 [70] 1 ICU	*N* = 40 Immuno- compromised – CPE: 22.5% – Hypercapnia 25%	NIV facemask (*n* = 20) vs. COT (*n* = 20)	*Intubation*: NIV 20% vs. COT 70% (*p* = 0.02) – Timing not specified *Mortality ICU*: NIV 20% vs. 50% (*p* = 0.05)
Martin, 2000 [71] 1 ICU	*N* = 61 Pneumonia – COPD: 38%	NIV facemask (*n* = 32) vs. COT (*n* = 29)	*Intubation*: NIV 28% vs. COT 59% (*p* = 0.02) – Timing not specified *Mortality ICU*: NIV 16% vs. 34% (*p* = 0.14)
Confalonieri, 1999 [72] 3 ICUs	*N* = 56 Acute hypoxemic RF – COPD: 41%	NIV facemask (*n* = 28) vs. COT (*n* = 28)	*Intubation*: NIV 21% vs. COT 50% (*p* = 0.03) – Timing: NIV 44 ± 24 h vs. COT 42 ± 13 h (p = NS) *Mortality hospital*: NIV 25% vs. 21% (p = NS)
Wood, 1998 [82] 1 ED	*N* = 27; Acute hypoxemic respiratory failure – CPE 37% - COPD 22%	NIV facemask (*n* = 16) vs. COT (*n* = 11)	*Intubation*: NIV 44% vs. COT 45% (*p* = 0.93) – Timing: NIV 26 ± 27 h vs. COT 4.8 ± 6.9 h (*p* = 0.055) *Mortality hospital*: NIV 25% vs. 0% (*p* = 0.12)
Kramer, 1995 [73] 1 ICU	*N* = 31; Acute hypoxemic respiratory failure – COPD: 74%	NIV facemask (*n* = 16) vs. COT (*n* = 15)	*Intubation*: NIV 31% vs. COT 73% (*p* = 0.03) – Timing not specified *Mortality hospital*: NIV 6% vs. 13% (p = NS)
Wysocki, 1995 [81] 1 ICU	*N* = 41; Acute hypoxemic respiratory failure – CPE 34% – Hypercapnia 41%	NIV facemask (*n* = 21) vs. COT (*n* = 20)	*Intubation*: NIV 62% vs. O_2_ 70% (*p* = 0.88) – Timing: NIV 16 ± 24 h vs. O_2_ 17 ± 25 h (*p* = 0.75) *Mortality ICU*: NIV 33% vs. O_2_ 50% (*p* = 0.46)

Values are given in mean ± standard deviation or median [25^ème^ – 75^ème^ percentiles]

**Abbreviations**: RCT = Randomized Controlled Trials; ICU = Intensive Care Unit; ED = Emergency Department; RF = Respiratory Failure; ARDS = Acute Respiratory Distress Syndrome; COPD = Chronic Obstructive Pulmonary Disease; CPE = Cardiogenic Pulmonary Edema; COT = Conventional Oxygen Therapy; HFNC = High-Flow Nasal Cannula oxygen therapy; NIV = Non-Invasive Ventilation; NS = Not Significant

### High-flow nasal cannula oxygen therapy (HFNC)

While COT cannot deliver FiO_2_ exceeding 60–70% in the upper airways [3], even with non-rebreathing facemask and especially in patients generating strong efforts, HFNC can achieve FiO_2_ levels exceeding 80–90% [36]. Beyond improving oxygenation and enhancing comfort, dead space washout of the upper airways and, to a lesser extent, continuous delivery of low levels of positive airway pressure can reduce work of breathing as compared with COT [37–39]. Indeed, relief of respiratory workload could be the main objective of noninvasive respiratory supports in severe forms of respiratory failure, possibly leading to improved outcomes [40]. The FLORALI study was the seminal study, showing clinical benefits of HFNC as compared to COT or NIV in patients with acute hypoxemic respiratory failure [1]. Whereas the risk of mortality was significantly lower with HFNC as compared with COT or NIV, the risk of intubation decreased only in patients with moderate-to-severe hypoxemia (PaO_2_/FiO_2_ ≤ 200 mm Hg), a finding suggesting that beneficial effects were more pronounced in patients with greater respiratory severity. In another large-scale clinical trial including immunocompromised patients, HFNC did not show any difference in terms of intubation or mortality as compared with COT [41] Another small-scale study reported lower intubation rates with HFNC than with COT [42]. Despite these contradictory results, clinical practice guidelines drawn up before the COVID-19 pandemic suggested the utilization of HFNC as opposed to COT or NIV in patients with acute hypoxemic respiratory failure, although this was only a conditional recommendation [43]. HFNC was widely used for management of respiratory failure during the COVID-19 pandemic. The first retrospective observational studies conducted in China and then in Europe suggested decreased risk of intubation with HFNC as compared with COT, while no reduction in mortality was observed [44–46]. After which, 6 clinical trials compared HFNC vs. COT in acute hypoxemic respiratory failure due to COVID-19 (Table 2) [47–52]. Two of them showed lower intubation rates with HFNC without reduction of mortality [47, 48], while a third one showed benefits on mortality or intubation but only in the subgroup of the most hypoxemic patients [49]. A recent meta-analysis pooling these randomized controlled trials confirmed that HFNC significantly reduced the risk of intubation compared with COT without changes in mortality rates in patients with respiratory failure due to COVID-19 [53]. However, early initiation of HFNC in the specific population with mild hypoxemia seems pointless, and HFNC should be considered mainly in patients with moderate-to-severe hypoxemia (PaO_2_/FiO_2_ ratio ≤ 200 mm Hg) [1, 51, 54]. A large-scale clinical trial (1110 patients planned to be included) comparing HFNC and COT is currently ongoing in patients with moderate-to-severe hypoxemia and will probably make it possible to reinforce or not the recommendation for the use of HFNC as first-line therapy in acute hypoxemic respiratory failure (NCT04468126).

### Continuous positive airway pressure (CPAP)

Whereas CPAP significantly improves oxygenation and increases end-expiratory lung volumes compared with COT and even HFNC, it has almost no effect on work of breathing in acute hypoxemic respiratory failure not related to cardiogenic pulmonary edema [55–57]. In 2000, a first clinical trial did not show any benefit of CPAP over COT (Table 3) [58]. Two other trials failed to show a decrease risk of intubation with CPAP delivered through a helmet vs. COT [59, 60]. Thereafter, a small-scale clinical trial including 40 patients with hematologic malignancy found that CPAP decreased the need for intubation and mortality when administered early in the ward as compared with COT [61]. More recently, a large platform trial conducted during the COVID-19 pandemic showed significantly lower intubation rates with CPAP through a facemask as compared with COT, a difference not observed between HFNC and COT [49]. This trial revived the interest in this noninvasive respiratory support which was nonetheless not as frequently used for management of respiratory failure [29]. However, another clinical trial conducted during the COVID-19 pandemic did not replicate these results, showing similar intubation and mortality rates for CPAP, COT or HFNC [50]. However, these recent studies have highlighted frequent discomfort using CPAP, leading to discontinuation of this respiratory support in approximately 15 to 20% of cases [49, 50]. Although discomfort may be lower when CPAP is delivered with a helmet, only a few small-scale clinical trials have compared CPAP-helmet vs. COT, with contradictory results [59–61].

### Noninvasive ventilation (NIV)

The early 1990s saw the first studies demonstrating the benefits of NIV through a facemask over COT in patients with acute exacerbation of chronic obstructive pulmonary disease [62–64] or cardiogenic pulmonary edema [65–67]. Later, in the 90–2000 s, several small-scale clinical trials involving patients with acute hypoxemic respiratory failure reported lower intubation rates with NIV compared COT (Table 4) [68–73]. From 2015, new clinical trials including larger populations (between 100 and more than 300 patients) compared NIV with HFNC [1, 74–79]. None of them reported beneficial effects of NIV with facemask as compared to HFNC [1, 74–78]. Two clinical trials have even shown deleterious effects of NIV with higher intubation or mortality rates than with HFNC [1, 77].

Whereas COT was used as control group in all the old studies showing beneficial effects of NIV [68–73, 80], HFNC was used in more recent studies as a control group [1, 74–79]. Given the potential superiority of HFNC over COT in reducing intubation [1, 43, 47, 48, 53], it is probably more difficult to show the superiority of NIV over HFNC than over COT. Moreover, the older studies included heterogeneous populations with a number of patients with underlying chronic lung disease or with cardiogenic pulmonary edema [68, 70–73, 81, 82], i.e. situations where NIV is particularly effective. By contrast, these patients were systematically excluded in more recent studies [1, 74, 76–79]. Lastly, the number of included patients was markedly lower in older than in most recent studies, with results appearing particularly contradictory in immunocompromised patients [69, 70, 74, 78]. Whereas two clinical trials conducted in 2000s showed superiority of NIV over COT on a sample of 40–50 patients [69, 70], two large-scale trials conducted more recently and including approximately 300 patients did not find any superiority of NIV over COT or HFNC [74, 78].

The interface may also significantly impact the outcome of NIV. Whereas NIV is most frequently delivered in ICUs with a facemask, it may also be delivered with a helmet. Potential advantages of a helmet include delivering higher pressures (inspiratory and expiratory) than with a facemask due to fewer leaks, and more prolonged sessions of NIV due to a more comfortable interface without face pressure points [83]. Several physiological studies have shown that NIV through helmet improved oxygenation, decreased patient inspiratory effort and relieved dyspnea as compared to HFNC [57, 84]. A recent clinical trial showed lower rates of intubation with NIV through helmet than with HFNC in patients with acute hypoxemic respiratory failure due to COVID-19 [79]. Two other randomized controlled trials have compared NIV through helmet versus NIV through facemask with contradictory results [85, 86]. In a first trial including 83 patients, the risks of intubation and mortality significantly decreased with NIV-helmet as compared to NIV-facemask [85]. In a more recent trial including 320 patients with acute hypoxemic respiratory failure due to COVID-19, NIV-helmet did not decrease the risk of intubation or mortality as compared to usual respiratory supports that included NIV-facemask in approximately 70% of cases and HFNC in 75% of cases [86].

Thereby, this strategy cannot be recommended to date, and further studies are needed to assess the clinical efficacy of this interface. Indeed, even though NIV through helmet seems to be an effective noninvasive respiratory support in terms of oxygenation, work of breathing and relief of dyspnea, to date only one clinical trial has compared NIV through a helmet vs. HFNC [79]. However, a large-scale clinical trial (1200 patients planned to be included) comparing NIV through helmet, CPAP through helmet and HFNC in patients with acute respiratory failure is currently ongoing, and will probably make it possible to better specify the clinical benefits of each strategy, and the respective effects of the ventilation mode and the interface (NCT05089695).

Lastly, it has been suggested that the magnitude of inspiratory effort relief under NIV may be a good predictor of NIV success [40, 84]. However, measurement of inspiratory effort is not performed in daily practice and whether the escalation to NIV may be personalized according to a patient’s inspiratory effort remains to be determined [87].

### Which first-line noninvasive respiratory support should we propose in acute hypoxemic respiratory failure?

To summarize, HFNC seems superior over COT to avoid intubation and should probably be used as a first-line treatment in patients with acute hypoxemic respiratory failure requiring more than 6 L/min of oxygen (i.e. FiO_2_ at least 40%) or PaO_2_/FiO_2_ ≤ 200 mm Hg and a respiratory rate above 25 breaths per minute or clinical signs of respiratory distress, despite no benefits on mortality. Given that its beneficial effects on intubation remain uncertain, especially when compared with HFNC, CPAP cannot currently be recommended as a first-line of noninvasive respiratory support strategy in patients with acute hypoxemic respiratory failure. Despite older studies favoring NIV over COT, recent clinical trials fail to show beneficial effects with NIV as compared to HFNC. Although HFNC is easier to use than NIV, it is not available in all units, especially in emergency rooms. NIV or even CPAP may therefore be proposed as alternatives to COT in acute hypoxemic respiratory failure if HFNC is not available or in situations with constraints as was the case during the pandemic. By contrast, there is no evidence to support the use of NIV or CPAP as first-line treatment if HFNC is available. The main limitation of such recommendations could be their restricted applicability to low-income countries due to costs or constraints, the choice of the noninvasive respiratory support decided according to the availability on site.

### Is it the same in patients with do-not-intubate (DNI) order?

Initiation of a noninvasive respiratory support in patients with acute hypoxemic respiratory failure and a do-not-intubate order is frequent. In a systematic review of observational studies including more than 10,000 patients with acute respiratory failure treated with NIV or HFNC, the overall rate of do-not-intubate orders was 27% [88]. However, two clinical situations must be distinguished. The first is when there is a reasonable prospect of survival. In this case, the goal of the non-invasive respiratory support is hospital survival, even though a decision has been made to forgo intubation in case of respiratory worsening. The second case scenario is an end-of-life setting and the goal of the treatment is symptom alleviation and quality of dying. In the first situation, the choice should be the same as for patients with full resuscitation code leading to intubation in case of respiratory worsening. In a systematic review including more than 2,000 patients with a do-not-intubate order, overall survival rate was 56% at hospital discharge and 32% at 1-year [89]. Whereas hospital survival reached 68% for chronic obstructive pulmonary disease and cardiogenic pulmonary edema, it was only 41% for pneumonia, and 37% for patients with malignancy. Although few studies have evaluated the quality of life of survivors, it would not be altered when compared with baseline. In a prospective observational cohort study, the prevalence of anxiety, depression, and post-traumatic stress disorder-related symptoms in patients with do-not-intubate order were similar to those who were treated without do-not-intubate order [90].

In the end-of-life setting, a randomized controlled trial compared NIV vs. COT for management of acute respiratory failure in 200 patients with a life expectancy of less than 6 months [91]. Although dyspnea decreased more rapidly with NIV than with COT and morphine consumption was reduced, NIV was discontinued due to poor tolerance in 11% of cases, mainly related to mask intolerance and anxiety. NIV impairs the ability to communicate, and therefore, does not appear to be compatible with the psychological and spiritual needs of patients in this setting. Consequently, NIV cannot be recommended in terminally ill patients. Several clinical trials have shown that HFNC was superior to COT for dyspnea alleviation, and reduced dyspnea to the same extent as NIV [92–95], and several observational studies have used HFNC in first-line treatment as an alternative to NIV [96–98]. Given its good tolerance and its efficacy on dyspnea, HFNC could be considered as the first-line noninvasive respiratory support for management of acute respiratory failure in end-of life settings.

## Other treatments

Positioning and mobilization are part of the adjuvant treatments that can be offered in patients with acute hypoxemic respiratory failure.

### What are the potential beneficial effects of awake prone positioning (APP)?

As prone positioning has been shown to improve survival in intubated patients with acute respiratory distress syndrome (ARDS) [99], during the COVID-19 pandemic “awake” prone positioning (APP) was used early in non-intubated patients with acute respiratory failure [100–102]. These first observational studies showed significant improvement in oxygenation and reduction in respiratory rate without major complications. Following which, several clinical trials compared APP vs. usual care on the risk of intubation and mortality (Table 5) [103–109]. In these clinical trials, APP was started in ICUs or hospital wards, in patients treated with COT, HFNC or NIV, with a wide range of respiratory severity and APP duration. Consequently, intubation rates ranged from 10 to 40%. By pooling all RCTs, several meta-analyses showed that APP was associated with a significant decreased risk of intubation without improving survival [110, 111]. In fact, these findings are mainly driven by one large meta-trial showing a decreased risk of intubation [109]. In this meta-trial pooling 6 different RCTs conducted in six countries and including 1111 patients, a decreased risk of intubation was significant in only one participating country (Mexico) where APP sessions were much longer. A post-hoc analysis of this Mexican study suggested that APP sessions lasting at least 8 h/day were associated with treatment success [112]. With the exception of this study, no other randomized controlled trial has shown a significant reduction in the risk of intubation or mortality using APP. However, thanks to this large-scale positive study, meta-analyses are favor APP, with beneficial effects on oxygenation and on the risk of intubation, especially using prolonged APP sessions in the most severe patients [110]. The potential benefits of APP on the risk of intubation in patients with COVID-19 cannot be extrapolated to patients with another etiology of acute hypoxemic respiratory failure. Thus, further clinical trials are needed to assess APP in patients with acute hypoxemic respiratory failure from various causes.

**Table 5:** Main multicenter RCTs comparing awake prone positioning (APP) vs. standard position in acute hypoxemic respiratory failure due to COVID-19

Study, year *N* patients – centers	Noninvasive respiratory support and APP duration	Outcomes
Nay, 2023 (103) *N* = 267–12 centers	Under COT (96%) or HFNC: usual care (*n* = 132) vs. APP (*n* = 135) for 90 min/d [30–133]	*Intubation*: APP 7% vs. 10% (p = NS) *Mortality*: APP 0% vs. 3% (p = NS)
Alhazzani, 2022 (104) *N* = 400–21 centers	Under HFNC (70%), COT, or NIV: usual care (*n* = 195) vs. APP (*n* = 205) for 4.8 h/d [1.8-8.0]	*Intubation day-30*: APP 34% vs. 41% (p = NS) *Mortality day-60*: APP 22% vs. 24% (p = NS)
Fralick, 2022 (105) *N* = 248–15 centers	Under HFNC or NIV: usual care (*n* = 122) vs. APP (*n* = 126) for 6 h [1.5–12.8] within the first 72 h	*Intubation*: APP 5% vs. 4% (p = NS) *Mortality*: APP 1% vs. 1% (p = NS)
Gopalakrishnan, 2022 (106) *N* = 502–1 center	Room air or COT: usual care (*n* = 245) vs. APP (*n* = 257) for 4.3 h ± 2.9/d	*Intubation*: APP 10% vs. 10% (p = NS) *Mortality*: APP 16% vs. 15% (p = NS)
Qian, 2022 (107) *N* = 501–2 centers	Under COT (66%), NIV or HFNC: usual care (*n* = 243) vs. APP (*n* = 258) for 4.2 h/d [1.8–6.7]	*Intubation*: APP 12% vs. 12% (p = NS) *Mortality*: APP 21% vs. 23% (p = NS)
Rosén, 2021 (108) *N* = 75–3 centers	Under HFNC or NIV: standard (*n* = 39) vs. APP (*n* = 36) for 9.0 h/d [4.4–10.6]	*Intubation day-30*: APP 33% vs. 33% (p = NS) *Mortality day-30*: APP 17% vs. 8% (p = NS)
Ehrmann, 2021 (109) *N* = 1111–6 countries	Under HFNC: standard (*n* = 557) vs. APP (*n* = 564) for 5.0 h/d [1.6–8.8]	*Intubation day-28*: APP 33% vs. 40% (*p* = 0.004) *Mortality day-28*: APP 21% vs. 24% (p = NS)

Values are given in mean ± standard deviation or median [25^ème^ – 75^ème^ percentiles]

**Abbreviations**: RCT = Randomized Controlled Trials; APP = Awake Prone Positioning; COT = Conventional Oxygen Therapy; HFNC = High-Flow Nasal Cannula oxygen therapy; NIV = Non-Invasive Ventilation; NS = Not Significant

**Table 6:** Major and minor criteria for intubation proposed to the committee using Delphi method. The presence of only one major criterion should lead to consider immediate intubation whereas combination of several minor criteria should prompt intubation

Major criteria
- Cardiac or respiratory arrest
- Altered consciousness defined as a Glasgow coma scale < 9
- Persistent hypoxemia despite maximal oxygen delivery or maximal inspired fraction of oxygen (FiO_2_) defined as PaO_2_ < 60 mm Hg, PaO_2_/FiO_2_ < 60 mm Hg, or SpO_2_ < 88%
- Respiratory acidosis defined as pH < 7.20

Minor criteria
1. Clinical signs of Respiratory distress with increased accessory muscle activity
2. Increased respiratory rate > 30 breaths per minute
3. Persistent hypoxemia despite maximal oxygen delivery or maximal inspired fraction of oxygen (FiO_2_) defined as PaO_2_ < 100 mm Hg; PaO_2_/FiO_2_ < 100 mm Hg; or SpO_2_ < 92%
4. Episodes of oxygen desaturation defined as SpO_2_ < 86%
5. Intolerance to device delivering oxygen
6. Abundant secretions
7. Respiratory acidosis defined as pH < 7.30
8. Agitation
9. Altered consciousness defined as Glasgow coma scale < 12
10. Shock requiring with increased lactate level at least 2 mmol/L

### Is physiotherapy a beneficial adjuvant measure?

To date, no randomized controlled trial has assessed the impact of physiotherapy in patients with acute hypoxemic respiratory failure. However, some techniques can be proposed to improve the clinical condition or comfort of patients during the management of respiratory failure. Motor physiotherapy such as exercises in bed, sitting on a chair, cycloergometer could help to reduce the shunt effect caused by parenchymal consolidations through alveolar recruitment. Several studies have evaluated the effects of physical activity on lung aeration using electrical impedance tomography [113–115]. In these studies, however, the changes in ventilation distribution were not sustained over time and regressed after the various interventions. Guidelines from the American Association for Respiratory Care and the British Thoracic Society have specified the role of respiratory physiotherapy [116, 117]. Bronchial clearance techniques should be reserved for patients with bronchial congestion and sputum difficulties. Respiratory physiotherapy should be tailored rather than routinely offered to all patients with acute hypoxemic respiratory failure. Among the various techniques used for respiratory physiotherapy, none has been shown to be superior to another, and the choice of the technique must take into account the patient’s tolerance, preference and clinical condition [118]. However, the physiotherapist can legitimately participate in the installation and monitoring of devices such as aerosol therapy or non-invasive ventilation [119, 120]. In all cases, the benefit-risk balance should be evaluated before and during each physiotherapy session.

## Indications for invasive mechanical ventilation

### Patient self-inflicted lung injury associated with noninvasive respiratory supports

After several small-scale studies showing beneficial effects of NIV, a large-scale clinical trial including more than 300 patients showed for the first time higher mortality rates with NIV than with HFNC [1]. A post-hoc analysis of this study suggested that large tidal volumes generated by the patient from NIV initiation (exceeding 9.5 ml/kg of predicted body weight) were associated with increased risk of death [121]. This finding was consistent with another observational study [122], and that gave birth to the concept of “PSILI” for Patient Self-Inflicted Lung Injury [123]. Patients producing strong inspiratory efforts generate large tidal volumes under NIV, and that may lead to worsening of lung injury by increasing transpulmonary pressures, in the same way that large tidal volumes are harmful in patients with ARDS under invasive mechanical ventilation [124, 125]. However, such large tidal volumes observed in patients treated with NIV may simply reflect respiratory disease severity, and one cannot exclude the possibility that effects of NIV may be different according to severity. An observational study suggested that NIV may be associated with an increased risk of death in the most severe patients, i.e. patients with ARDS and a PaO_2_/FiO_2_ ratio below 150 mm Hg [126]. Similarly, another observational study showed that patients who still had significant inspiratory efforts after NIV initiation had increased risk of intubation as compared to the others [40]. Although tidal volumes do not increase when switching form COT to HFNC [37], patients with strong inspiratory efforts could still develop lung injury, regardless of the type of noninvasive respiratory support [123]. Up until now, no clinical trial has shown any benefit to switch from a noninvasive respiratory support to another according to tidal volumes or intensity of effort.

### Criteria for intubation

Most clinical trials comparing different noninvasive respiratory supports have proposed pre-specified criteria for intubation to ensure the consistency of indications across sites and reduce the risk of delayed intubation. Criteria for intubation usually include worsening respiratory failure, hemodynamic failure and neurological failure. Major and minor criteria for intubation were determined by experts using a Delphi method and proposed to the guideline panel (Table 6). The presence of only one major criterion should lead to consider immediate intubation whereas a combination of several minor criteria should prompt intubation.

### Timing of intubation

Several observational studies have suggested that late or delayed intubation may be associated with increased risk of death [127–130]. However, there may be a major bias of interpretation between late intubation and delayed intubation. Delayed intubation means a delay between occurrence of criteria for intubation and the decision to intubate. Late intubation can occur without being delayed, for example when criteria for intubation emerge later due to secondary worsening. An observational study including more than 800 patients treated with NIV has showed that patients intubated early (i.e. within the first 12 h after ICU admission) had markedly higher severity at admission than those intubated later [128]. However, the severity assessed at the time of intubation was similar for both early and late intubations, meaning that intubation was late but not delayed. Thereby, late intubation could be associated with worse outcomes simply because it indicates failure of the initial treatment. Randomized controlled trials are the best way to answer the question of whether the use of noninvasive respiratory supports risks delaying intubation. Among all randomized controlled trials that compared HFNC vs. COT, none showed significantly later intubation with HFNC than with COT (Table 2). Similarly, among all randomized controlled trials that compared NIV vs. HFNC or COT, none showed significant later intubation than with one of those respiratory supports (Table 4). Only one RCT showed later intubation with CPAP than with COT in patients with respiratory failure due to COVID-19, knowing that 40% of patients were treated in the hospital wards and not in ICUs due to a wave of the pandemic (Table 3) [49]. Therefore, although late intubation per-se may be associated with poor outcomes, randomized controlled trials do not show that noninvasive respiratory supports may lead to late or delayed intubation.

### Where to manage patients receiving noninvasive respiratory support?

The huge influx of ICU patients during the COVID-19 pandemic led intensivists to treat a large number of patients with noninvasive respiratory supports outside ICUs due to the limited number of available beds [46, 49, 131]. These patients were treated in wards with HFNC, CPAP or NIV, and only those requiring intubation were admitted to an ICU, with intubation rates around 30–40% [49]. In an observational study including 608 patients in 10 hospitals in the Netherlands during the pandemic, initiation of HFNC outside ICUs was shown to be safe, and intubation or mortality rates did not differ between patients treated first in ICUs and those treated first outside ICUs [132]. In a French observational study, 85 patients with acute hypoxemic respiratory failure due to COVID-19 received open valve CPAP treatment in intermediate care units from non-ICU staff who were trained using a simple short tutorial video [133]. In a retrospective study conducted in Italy before the pandemic, patients were treated with CPAP or NIV outside ICUs without major complication [134]. However, these patients were managed by a rapid response team with a daily visit in collaboration with ward staff highly experienced in noninvasive respiratory supports. The pandemic has shown that initiation of noninvasive respiratory support outside ICUs was feasible and potentially safe for patients with respiratory failure, especially when the hospital faces such constraints. However, intubation rates in patients with acute hypoxemic respiratory failure range from 30 to 50%, and in more than half of cases occur within the first 24 h (Tables 2 and 3 and Table 4). Therefore, these patients should be closely monitored in ICUs rather than in wards, if ICU beds are available.

## Conclusion

Oxygen supplementation should be initiated for patients with acute hypoxemic respiratory failure defined as PaO_2_ below 60 mm Hg or SpO_2_ < 90% on room air. HFNC should be the first-line noninvasive respiratory support in patients with moderate-to-severe hypoxemia (PaO_2_/FiO_2_ ≤ 200 mm Hg). Further studies are needed to assess potential benefits of CPAP, NIV through a helmet and awake prone position, especially in patients with acute hypoxemic respiratory failure not related to COVID-19.

## Data Availability

Not applicable.

## Abbreviations

RCT: Randomized Controlled Trials
COT: Conventional Oxygen Therapy
HFNC: High-Flow Nasal Cannula oxygen therapy
NIV: Non-Invasive Ventilation
ICU: Intensive Care Unit
NS: Not Significant
